# Risodontropy

**Published:** 1855-01

**Authors:** S. D. Muse

**Affiliations:** Monticello, Miss.


### RISODONTRYPY.

BY S. D. MUSE, D. D. S.

OF MONTICELLO, MISS.

MR. EDITOR:

"Honor to whom honor is due," is an old maxim, but not the less deserving of being observed and practiced upon on that account. If this be true, it is but just that the author of any and every discovery or invention calculated to advance the science of Dental Surgery should be traced to its legitimate source, that such author may receive, from a liberal profession, the mead of praise, thanks, and reputation due for the same.

The constant reader of "Dental Literature" will be able to call to mind not a few instances in which scientific, investigating dental practitioners have been, or attempted to be, robbed of invaluable inventions and discoveries. And in all these, or at least in a number of instances, those claiming originality have, or profess to have, practiced "secretly," until the publication of the real discoverer or inventor. When in they come setting up a claim to that which they have no title, whatever. Indeed, had they conceived a similar idea and kept it "secret," that alone is, or should be amply sufficient to deprive them of all claim to such invention, or discovery, and will at least shake the confidence of the profession in their pretentions to title.

I hold that *it is* unprofessional for any one member of the dental profession to conceal from the profession any invention or discovery practically useful to the profession, or to those who are so unfortunate as to require the services of the dental practitioner.

I am aware of apparent digression. This, however, may be pardoned in one who seldom writes at all, and never attempts to embellish anything, but simply to state, in all candor, such facts as may present themselves, and drop them for what they are worth, or will go for.

We are undoubtedly indebted to our indefatigable, ingenious, and investigating contemporary, Dr. S. P. Hullihen, for the discovery of the operation under consideration. I am well aware that others have set up claim, but have they or can they establish this claim? I am fully persuaded they *can not*.

I am well apprised, from the position I have taken, that I may be charged with meddling with the business of others.

If so, I am willing to take the consequences. I shall contend, however, that although I do not allege to having been robbed myself of either invention or discovery. This is the business of every member of the profession, to render to "Cæsar that which is Cæsar's. Thereby encourage every one to investigate, and promptly to impart to the fraternity the successful results of such investigation. Enough of this. There can now be but little doubt of the utility and success of this operation in very many cases. Success, however, will depend much upon the age, temperament, and health of the patient, also on the peculiar organization of the teeth. I have found but little difference in the success of the operation in the different class of the teeth in the same mouth, succeeding about as well in the molars, as bicuspide in the latter as the incisors. I use the same drill for performing the operation in all, and it is not sufficiently large to excise the nerve in any, yet the effect is the same. I have found the operation more successful in patients above twenty years old than those below that age. Indeed, I seldom perform it on those under that age, and still more so, in those of above twenty-five years old. And in teeth of considerable density of structure than those of greater vascularity. I never attempt the operation when there is much general nervous irritability. Or when the health of my patient is, and has for some time been delicate, or where there is much disease and irritation of the gums from any cause whatever.

I have not seen any satisfactory explanation of the *modus operandi* of this operation. It is asserted by some, that the nerve is severed by the drill. This, however, I *know* is not the case in my practice. My drill not being large enough to sever the nerve in an incision. Others contend that the result of wounding the nerve is inflammation and ultimate death of that organ. This last is equally fallacious as the first. We know that inflammation of the dental pulp is always, or nearly so, attended with pain, more or less severe, and usually of no very short duration; hence, the latter theory cannot be correct. Nor have I a better than either to offer. All I know is, that after the first shock there is but little further complaint on the part of the patient, either in excavating or filling, or in excising the dental pulp. There is, however, generally, some uneasiness the first night after the operation, but not sufficient to prevent sleep, after which, some soreness of the gum is the only inconvenience, and in no case have I seen extensive inflammation of the organs.

I will only remark that I have, in a few instances introduced the drill the day following the operation, for the purpose of drawing off the blood, which seldom or never fails to give entire relief.

The drill I use is made of an old steel excavator. I never use the bow, believing it less safe, and more painful.

NOTE. I am aware the perforating the fang has been recommended, and practiced long since, but for different purposes than for which the operation of Risodontrypy is performed. Heretofore, it was performed for the purpose of giving vent to matter secreted in the pulp cavity, or for the purpose of curing toothache, as discovered and practiced by M. Fatore.

